# Correction: Structure-selected RBM immunogens prime polyclonal memory responses that neutralize SARS-CoV-2 variants of concern

**DOI:** 10.1371/journal.ppat.1013707

**Published:** 2025-11-25

**Authors:** Gonzalo Almanza, Alex E. Clark, Valentina Kouznetsova, Eduardo Olmedillas, Andrea Castro, Igor F. Tsigelny, Yan Wu, George F. Gao, Sandra L. Leibel, William Bray, Erica O. Saphire, Aaron F. Carlin, Maurizio Zanetti

The initials of the eleventh author are indexed incorrectly in PubMed. The correct initials are: Saphire, E.O.
