# Supplementary figures and images for: Structure-selected RBM immunogens prime polyclonal memory responses that neutralize SARS-CoV-2 variants of concern
Source: PLoS Pathog. 2022 Jul 21;18(7):e1010686. doi: 10.1371/journal.ppat.1010686 (PMC9302722; doi:10.1371/journal.ppat.1010686)

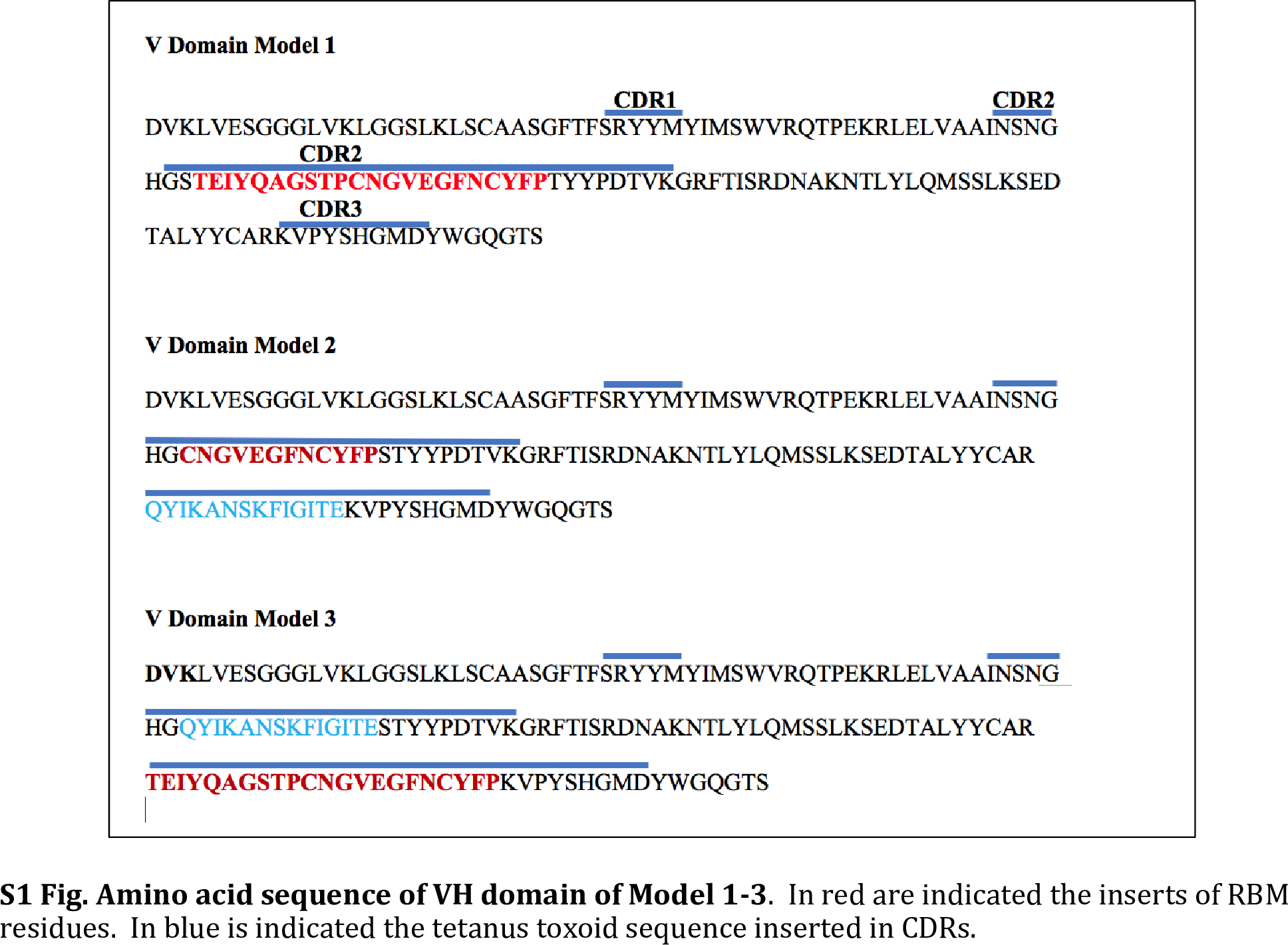

Supplement: S1 Fig — In red are indicated the inserts of RBM residues. In blue is indicated the tetanus toxoid sequence inserted in CDRs. (TIF) [file ppat.1010686.s001.tif]

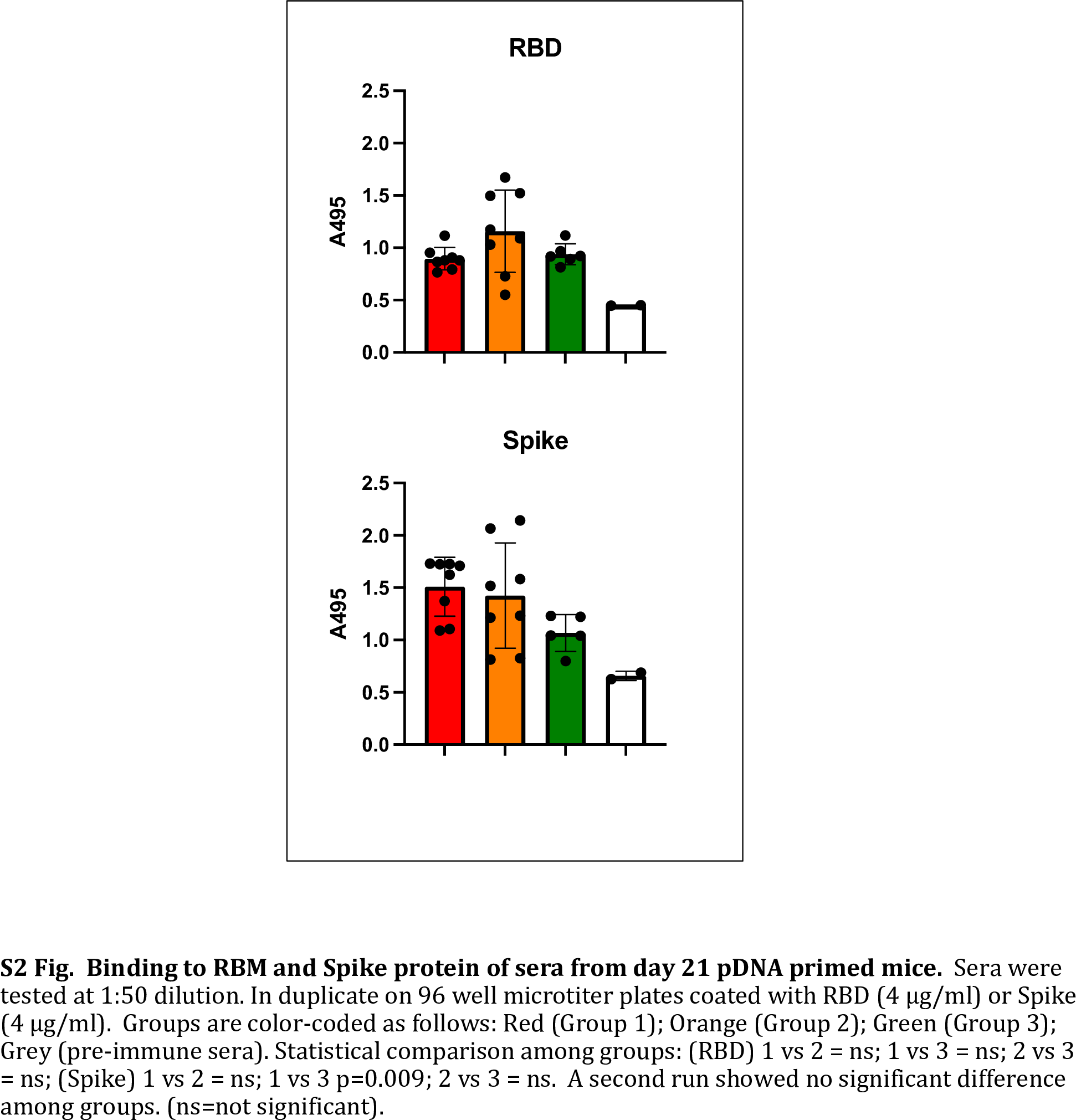

Supplement: S2 Fig — Sera were tested at 1:50 dilution. In duplicate on 96 well microtiter plates coated with RBD (4 μg/ml) or Spike (4 μg/ml). Groups are color-coded as follows: Red (Group 1); Orange (Group 2); Green (Group 3); Grey (pre-immune sera). Statistical comparison among groups: (RBD) 1 vs 2 = ns; 1 vs 3 = ns; 2 vs 3 = ns; (Spike) 1 vs 2 = ns; 1 vs 3 p = 0.009; 2 vs 3 = ns. A second run showed no significant difference among groups. (ns = not significant). (TIF) [file ppat.1010686.s002.tif]

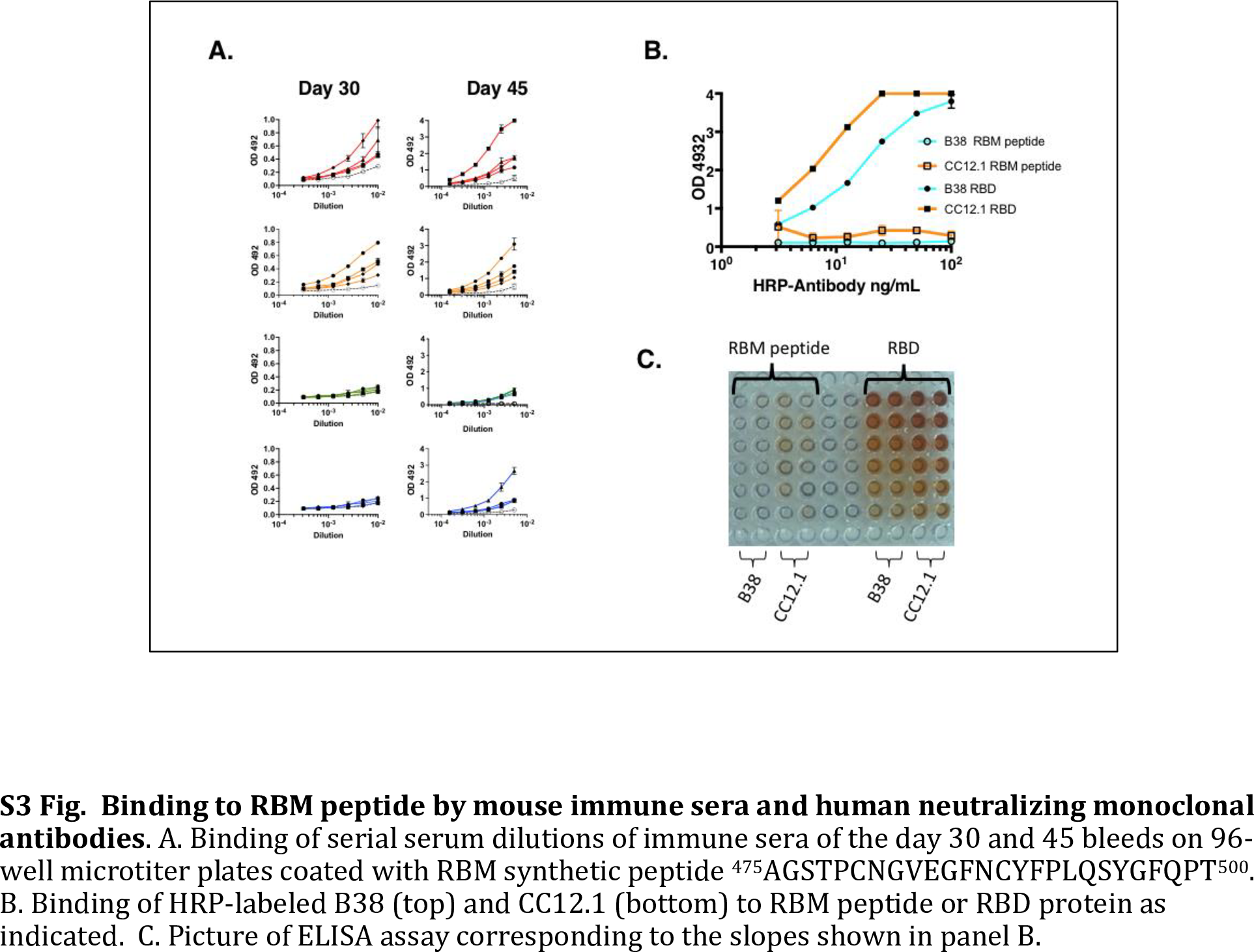

Supplement: S3 Fig — A. Binding of serial serum dilutions of immune sera of the day 30 and 45 bleeds on 96-well microtiter plates coated with RBM synthetic peptide 475AGSTPCNGVEGFNCYFPLQSYGFQPT500. B. Binding of HRP-labeled B38 (top) and CC12.1 (bottom) to RBM peptide or RBD protein as indicated. C. Picture of ELISA assay corresponding to the slopes shown in panel B. (TIF) [file ppat.1010686.s003.tif]

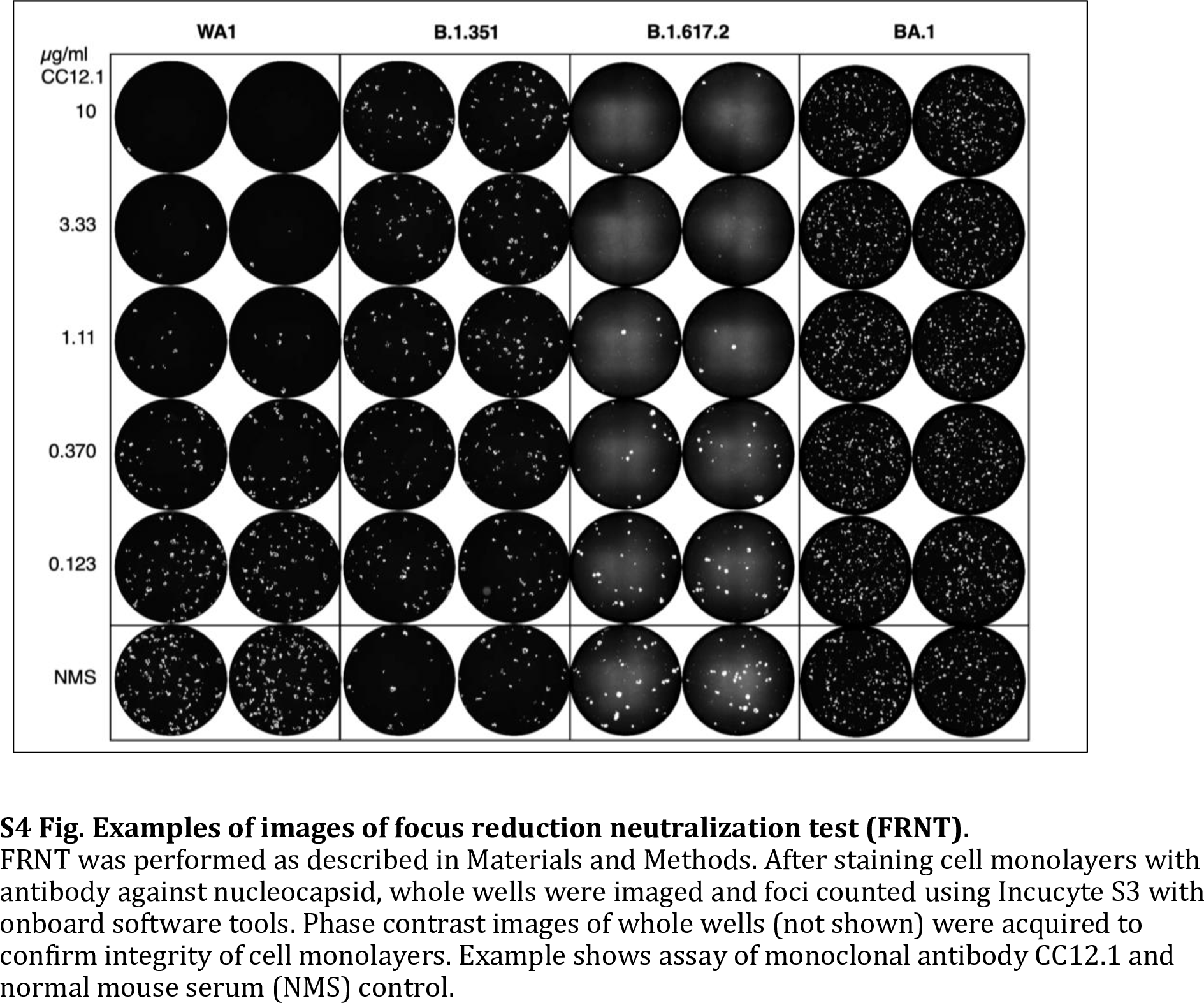

Supplement: S4 Fig — FRNT was performed as described in Materials and Methods. After staining cell monolayers with antibody against nucleocapsid, whole wells were imaged and foci counted using Incucyte S3 with onboard software tools. Phase contrast images of whole wells (not shown) were acquired to confirm integrity of cell monolayers. Example shows assay of monoclonal antibody CC12.1 and normal mouse serum (NMS) control. (TIF) [file ppat.1010686.s004.tif]

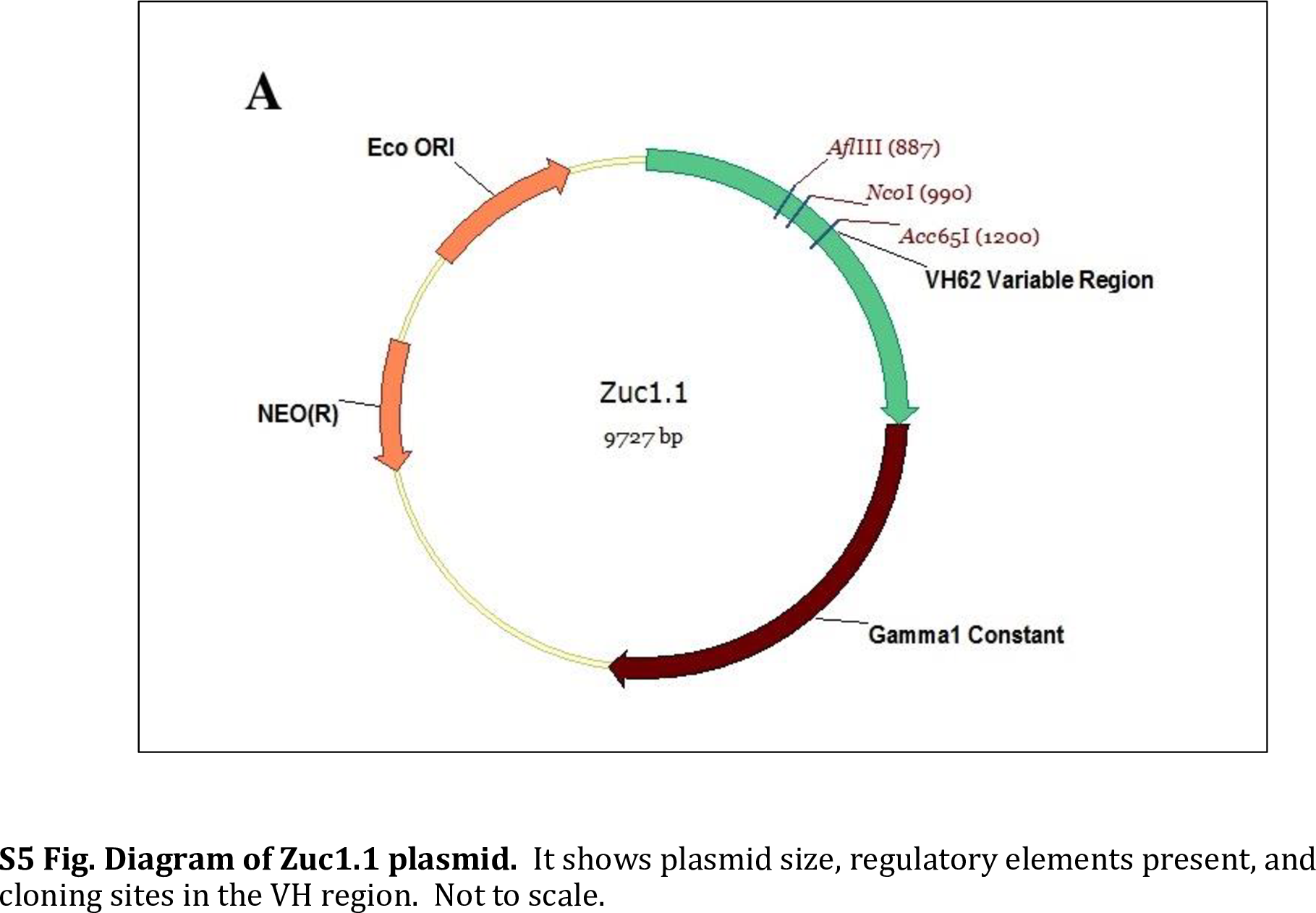

Supplement: S5 Fig — It shows plasmid size, regulatory elements present, and cloning sites in the VH region. Not to scale. (TIF) [file ppat.1010686.s005.tif]

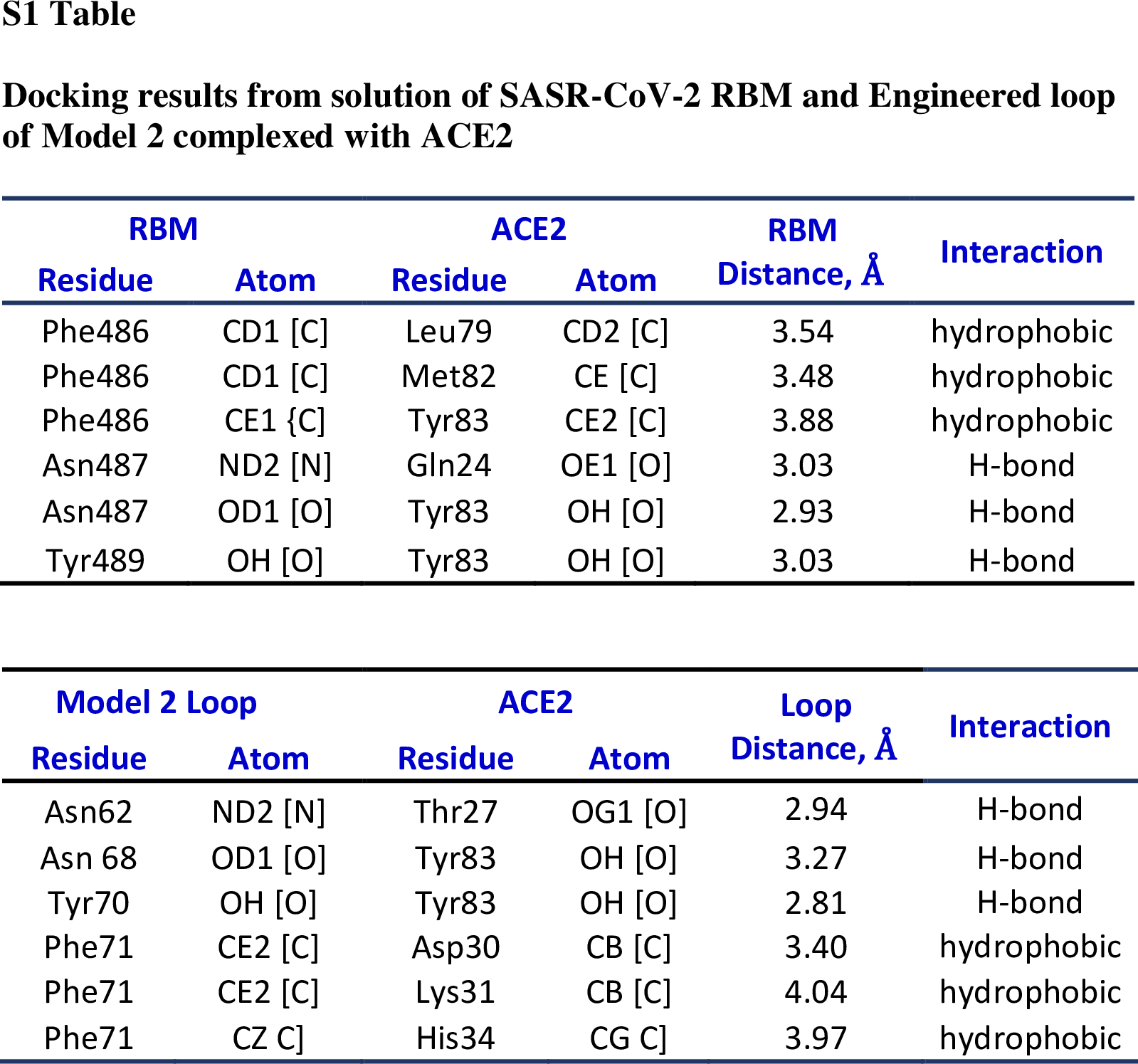

Supplement: S1 Table — (TIF) [file ppat.1010686.s006.tif]
